# Supplementary material for: The Association between Menstrual Symptoms and Presenteeism: A Cross-Sectional Study for Women Working in Central Tokyo
Source: Int J Environ Res Public Health. 2024 Mar 8;21(3):313. doi: 10.3390/ijerph21030313 (PMC10970697; doi:10.3390/ijerph21030313)
Supplement: Supplementary file 1 [file ijerph-21-00313-s001.zip › ijerph-2863290-supplementary.pdf]

Supplemental Table S1. Logistic regression analysis of the existence of frequently severe PMS or symptoms during menstruation and relative presenteeism (N=232)

Existence of frequently severe menstrual symptoms	Regression coefficient	SE	p value	Odds ratio	95% confidence interval
Group without severe PMS or menstrual symptom	ref	ref	ref	ref	ref
Group with any of the symptoms of severe PMS alone	-2.0296	0.3799	<0.01	0.13	0.06-0.27
Group with any of the severe symptoms during menstruation alone	-1.8389	0.4866	<0.01	0.16	0.06-0.41
Group with both severe PMS and symptoms during menstruation	-1.9567	0.3904	<0.01	0.14	0.06-0.30
